# Supplementary material for: Bayesian refinement of protein structures and ensembles against SAXS data using molecular dynamics
Source: PLoS Comput Biol. 2017 Oct 18;13(10):e1005800. doi: 10.1371/journal.pcbi.1005800 (PMC5662244; doi:10.1371/journal.pcbi.1005800)
Supplement: S1 Archive — (BZ2) [file pcbi.1005800.s011.tar.bz2 › gromacs_4.62_bayesian_saxs_refi/share/html/online/g_vanhove.html]

g\_vanhove


| g\_vanhove Main Table of Contents |

**VERSION 4.6  
Sat 19 Jan 2013**

---

### Description

g\_vanhove computes the Van Hove correlation function.
The Van Hove G(r,t) is the probability that a particle that is at r\_0
at time zero can be found at position r\_0+r at time t.
g\_vanhove determines G not for a vector r, but for the length of r.
Thus it gives the probability that a particle moves a distance of r
in time t.
Jumps across the periodic boundaries are removed.
Corrections are made for scaling due to isotropic
or anisotropic pressure coupling.

With option -om the whole matrix can be written as a function
of t and r or as a function of sqrt(t) and r (option -sqrt).

With option -or the Van Hove function is plotted for one
or more values of t. Option -nr sets the number of times,
option -fr the number spacing between the times.
The binwidth is set with option -rbin. The number of bins
is determined automatically.

With option -ot the integral up to a certain distance
(option -rt) is plotted as a function of time.

For all frames that are read the coordinates of the selected particles
are stored in memory. Therefore the program may use a lot of memory.
For options -om and -ot the program may be slow.
This is because the calculation scales as the number of frames times
-fm or -ft.
Note that with the -dt option the memory usage and calculation
time can be reduced.

### Files

| option | filename | type | description |
| --- | --- | --- | --- |
| **-f** | traj.xtc | Input | Trajectory: xtc trr trj gro g96 pdb cpt |
| **-s** | topol.tpr | Input | Structure+mass(db): tpr tpb tpa gro g96 pdb |
| **-n** | index.ndx | Input, Opt. | Index file |
| **-om** | vanhove.xpm | Output, Opt. | X PixMap compatible matrix file |
| **-or** | vanhove\_r.xvg | Output, Opt. | xvgr/xmgr file |
| **-ot** | vanhove\_t.xvg | Output, Opt. | xvgr/xmgr file |

### Other options

| option | type | default | description |
| --- | --- | --- | --- |
| **-[no]h** | bool | no | Print help info and quit || **-[no]version** | bool | no | Print version info and quit || **-nice** | int | 19 | Set the nicelevel || **-b** | time | 0 | First frame (ps) to read from trajectory || **-e** | time | 0 | Last frame (ps) to read from trajectory || **-dt** | time | 0 | Only use frame when t MOD dt = first time (ps) || **-[no]w** | bool | no | View output .xvg, .xpm, .eps and .pdb files || **-xvg** | enum | xmgrace | xvg plot formatting: xmgrace, xmgr or none || **-sqrt** | real | 0 | Use sqrt(t) on the matrix axis which binspacing # in sqrt(ps) || **-fm** | int | 0 | Number of frames in the matrix, 0 is plot all || **-rmax** | real | 2 | Maximum r in the matrix (nm) || **-rbin** | real | 0.01 | Binwidth in the matrix and for -or (nm) || **-mmax** | real | 0 | Maximum density in the matrix, 0 is calculate (1/nm) || **-nlevels** | int | 81 | Number of levels in the matrix || **-nr** | int | 1 | Number of curves for the -or output || **-fr** | int | 0 | Frame spacing for the -or output || **-rt** | real | 0 | Integration limit for the -ot output (nm) || **-ft** | int | 0 | Number of frames in the -ot output, 0 is plot all |

---

http://www.gromacs.org  
gromacs@gromacs.org
